# Mechanisms Underlying the Emergence of Post-acidosis Arrhythmia at the Tissue Level: A Theoretical Study

**DOI:** 10.3389/fphys.2017.00195

**Published:** 2017-03-30

**Authors:** Jieyun Bai, Renli Yin, Kuanquan Wang, Henggui Zhang

**Affiliations:** ^1^School of Computer Science and Technology, Harbin Institute of TechnologyHarbin, China; ^2^State Key Laboratory of Urban Water Resource and Environment, Harbin Institute of TechnologyHarbin, China; ^3^Biological Physics Group, School of Physics and Astronomy, University of ManchesterManchester, UK; ^4^Space Institute of Southern ChinaShenzhen, China

**Keywords:** post acidosis arrhythmias, ventricular tachycardia, premature ventricular complexes, delayed afterdepolarization, transmural dispersion of repolarization, sink-source mismatch

## Abstract

Acidosis has complex electrophysiological effects, which are associated with a high recurrence of ventricular arrhythmias. Through multi-scale cardiac computer modeling, this study investigated the mechanisms underlying the emergence of post-acidosis arrhythmia at the tissue level. In simulations, ten Tusscher-Panfilov ventricular model was modified to incorporate various data on acidosis-induced alterations of cellular electrophysiology and intercellular electrical coupling. The single cell models were incorporated into multicellular one-dimensional (1D) fiber and 2D sheet tissue models. Electrophysiological effects were quantified as changes of action potential profile, sink-source interactions of fiber tissue, and the vulnerability of tissue to the genesis of unidirectional conduction that led to initiation of re-entry. It was shown that acidosis-induced sarcoplasmic reticulum (SR) calcium load contributed to delayed afterdepolarizations (DADs) in single cells. These DADs may be synchronized to overcome the source-sink mismatch arising from intercellular electrotonic coupling, and produce a premature ventricular complex (PVC) at the tissue level. The PVC conduction can be unidirectionally blocked in the transmural ventricular wall with altered electrical heterogeneity, resulting in the genesis of re-entry. In conclusion, altered source-sink interactions and electrical heterogeneity due to acidosis-induced cellular electrophysiological alterations may increase susceptibility to post-acidosis ventricular arrhythmias.

## Introduction

Ischaemic heart disease is the leading cause of sudden cardiac death and reperfusion is the treatment to reduce the size of a myocardial infarction in patients (Carden and Granger, 2000). The myocardial injury paradoxically occurs with the acute reperfusion of ischaemic myocardium (Brooks et al., 1995). It has been suggested that reperfusion-induced arrhythmias, one form of myocardial reperfusion injury, are mainly due to the acute acidosis recovery (Nagai et al., 2010; Niwano and Tojo, 2010). In fact, gradual reperfusion induced by ischaemic postconditioning, has been shown to delay restoration of intracellular pH and prevent reperfusion-induced ventricular tachycardia (VT) and ventricular fibrillation (VF) (Avkiran et al., 1996). Therefore, identifying the mechanisms underlying the development of VT/VF in acidotic settings is important for clinical practices to restore intracellular pH during reperfusion (Vandenberg et al., 1993; Kapur et al., 2009; Nagai et al., 2010). However, it is difficult to identify such mechanisms due to fast change of substrates involved in ventricular arrhythmias during ischaemia-reperfusion injury (IRI) in patients. Although previous animal studies have suggested that VT might arise from focal and/or reentrant activities (Boineau and Cox, 1973; Orchard and Cingolani, 1994; Pogwizd et al., 1998; Jie et al., 2010), the precise mechanisms leading to increased arrhythmic risk in the IRI remain incompletely understood.

A marked acidosis occurs during myocardial ischaemia (Park et al., 1999), which may play a crucial role in the arrhythmogenesis during IRI. Experimental studies have suggested that post-acidosis arrhythmias during acute reperfusion are mainly triggered by delayed afterdepolarizations (DADs) at the cellular level (Said et al., 2008; Lascano et al., 2013). DADs are membrane depolarization of cardiac myocytes that appear following the repolarization of the action potential (AP), and are possibly related to the high frequency of spontaneous calcium sparks produced by overloaded calcium in the sarcoplasmic reticulum (SR) (Mészáros et al., 2001). Although the calcium overloaded SR was observed during acidosis, the DADs occurred after acidosis (Said et al., 2008). The explanation for this phenomenon may be given by the experimental evidence showing the phosphorylation of the Thr^17^ site of phospholamban (PLN) due to calcium/calmodulin-dependent protein kinase II (CaMKII) activation (Said et al., 2008) and the inhibitory effects of acidosis on ryanodine receptor 2 (RyR2) (Balnave and Vaughan-Jones, 2000) and sodium-calcium exchanger (NCX) (Terracciano and MacLeod, 1994). In particular, the enhanced effects of CaMKII activation may offset the direct inhibitory effect of acidosis on calcium-ATPase 2a (SERCA2a) and therefore the calcium overloaded SR during acidosis (Lascano et al., 2013). Further studies have showed that gradual reperfusion instead of acute reperfusion can delay recovery of acidosis and protect against ventricular arrhythmogenesis (Avkiran et al., 1996; Kin et al., 2004; Inserte et al., 2009). From these results it is reasonable to expect that the abrupt pH restoration may produce spontaneous SR calcium release and the consequent DADs, thus providing a potential mechanistic link between IRI and ventricular arrhythmogenesis. However, the DADs observed in isolated cells cannot be extrapolated directly to focal arrhythmias in the intact heart. This connection cannot be made because the capability of DADs to evolve into focal arrhythmias depends on their ability to spread into surrounding neighbor cells. In this case, cells with DADs act as a “source” of excitation to drive the surrounding quiescent myocardium, and the surrounding myocardium acts as a “sink” that would suppress the “excitation source” because of intercellular electrotonic interaction. Therefore, for localized DADs to propagate, it requires two conditions: (1) each cell in the DAD region (acting as a “source”) must reach its activation threshold and initiate an action potential (AP), and (2) cells in the surrounding tissue (acting as a “sink”) must receive sufficient stimulus current flowing from the “source” through intercellular gap junctional coupling to be excited (Spector, 2013). Thus, important questions need to be answered, such as the extent to which the SR calcium load is required to depolarize the cell in the “source” region to trigger an AP; and the number of DAD cells required to overcome the source-sink mismatch (i.e., insufficient “source” to drive the “sink”) between an acidotic zone and the surrounding regions to produce a PVC in cardiac tissues.

In addition to DADs in single myocytes, life-threatening VT/VF in intact hearts of patients during IRI also was observed (Tsujita et al., 2004). When VT/VF is initiated, reentry of the propagation occurs and forms a spiral wave in tissues. Despite its importance in clinical settings, a clear comprehension of the mechanisms underlying re-entrant arrhythmia in post acidosis at tissue level is lacking. Although it is known that changes in AP and cytoplasmic calcium concentration induced by acidosis may constitute arrhythmogenic substrates for reentry, the evolution of reentry resulted from acidosis-induced electrophysiological changes has not yet been fully characterized. Multiple experimental studies demonstrated that increased cytoplasmic calcium concentration may increase gap junction resistance (Noma and Tsuboi, 1987; Peracchia, 2004), and down-regulation of connexin proteins as well as the presence of severe fibrosis were also observed in ischaemic tissues (de Groot et al., 2001; de Groot and Coronel, 2004; Sánchez et al., 2011; Saffitz and Kleber, 2012). Cell-to-cell uncoupling would be expected to lead to slow propagation of excitation waves (Saffitz and Kleber, 2012). Moreover, different laboratories have shown that acidosis causes heterogeneous changes (prolongation /abbreviation) of APs (Levites et al., 1975; Bethell et al., 1998; Komukai et al., 2002; Kazusa et al., 2014), resulting in altered electrical heterogeneity in tissues and therefore dispersion of repolarization (TDR), increasing susceptibility to re-entrant arrhythmias (Kuo et al., 1983; Laurita and Rosenbaum, 2000; Bernus et al., 2005; Qu et al., 2006; Jie et al., 2008; Jie and Trayanova, 2010). Whereas the development of reentry has been demonstrated to be associated with slow conduction and TDR (Boineau and Cox, 1973; Kuo et al., 1983), this association is not clear for the gradual evolution from DADs at the cellular level to PVCs and reentry at the tissue level in post acidosis arrhythmias.

Modeling studies have shed light on the mechanisms of post-acidosis arrhythmias. Based on experimental data on acid-sensing ion channels, computational models provided physiological insights into the relationship between acidosis-induced changes in electrophysiological properties and ventricular arrhythmogenesis (Crampin and Smith, 2006; Crampin et al., 2006; Roberts and Christini, 2011, 2012; Lascano et al., 2013). In single-cell simulations, the pro-arrhythmic role of CaMKII activation (Lascano et al., 2013) and sodium-potassium pump (Roberts and Christini, 2011) have been demonstrated, respectively, as they contribute to an increase in cytoplasmic calcium concentration and a rise in intracellular sodium concentration, which may contribute to the genesis of DADs after acidosis. The emergence of arrhythmias arising from DADs induced by reperfusion remains lacking. Characterizing this evolution, through the use of multi-scale models from subcellular, cellular, tissue, organ to system levels, has the potential to help understand better the mechanisms underlying the reperfusion induced arrhythmias in IRI.

The main objective of the present work is to explore the mechanisms underlying the emergence of post-acidosis arrhythmia. For this purpose, single cell, 1D tissue strand and 2D tissue sheet simulations were performed to investigate the functional impacts of acidotic conditions on the electrical activity, with a particular focus on the genesis of spiral waves in the ventricular transmural wall. These models were used to study: (1) the quantitative relationship between the SR calcium content and the DAD amplitude; (2) the DAD amplitude required to reach the threshold for triggering an AP; (3) the recovery time of intracellular acidosis that influences the protection from the occurrence of DADs; (4) the individual contribution of the SR calcium load and the gap junction uncoupling to the enhanced source-sink interactions for producing a PVC; and (5) the effect of electrical heterogeneity on the vulnerability of tissue to unidirectional conduction block that facilitates the initiation of re-entry. These results may provide insights into the evolution of reperfusion-induced VT.

## Materials and methods

### Model of DADs in single cardiac myocytes

To model the electrical excitation behavior of cardiac myocytes, well-characterized AP models developed by ten Tusscher et al. (TP06 model) were used (ten Tusscher and Panfilov, 2006). These models are chosen because they are not only based on available human ventricular data but also reproduce the ion channel kinetics and membrane potentials of human ventricular cells. Most importantly, these models incorporated transmural heterogeneity in ventricular electrophysiology to reproduce APs of human epicardial (Epi), mid-myocardial (Mid) and endocardial (Endo) cells. These models have been suggested to be suited for the study of spiral wave dynamics in human ventricular tissues (ten Tusscher et al., 2009).

To reproduce cardiac electrical behavior under acidotic and post acidotic conditions, some modifications to the TP06 models were included. Specifically, the calcium release from the SR (Irel) was modeled as a single flow, which combined the actions of calcium-induced-calcium release from the SR and calcium leak through RyR2 (Lascano et al., 2013). Intracellular pH regulation (Crampin and Smith, 2006) and CaMKII activation (Decker et al., 2009) were also introduced into TP06 cell models. Details can be found in Supplementary Information.

The protocol of pH changes (PPC) similar to that in a previous study (Lascano et al., 2013) was used to perform single cell simulations. PPC consisted of a 1-min-long control period, a 6-min-long acidosis period and a 5-min-long post acidosis period. Intracellular pH was set to 6.7 during the acidosis period and was set to 7.15 during the control period and post acidosis period. Single cells were paced with a constant pacing frequency of 70 beats/min. This PPC was used to predict the triggering of DADs. Changes in fraction of activated CaMKII (CaMK_active_), maximum cytoplasmic calcium concentration ([Ca^2+^]_i_(max)), maximum SR calcium concentration ([Ca^2+^]_SR_(max)), maximum intracellular sodium concentration ([Na^+^]_i_(max)) and the NCX current (INCX) in the PPC were used to study impairment of calcium handling. The time courses of voltage waveforms, underlying INCX, Irel, cytoplasmic calcium concentration ([Ca^2+^]_i_) as well as SR calcium concentration ([Ca^2+^]_SR_) were analyzed to investigate the effect of post acidosis in genesis of DADs. The occurrence time of DADs in the single cells was calculated as the time interval between the beginning time of the last stimulus during acidosis and the time when the first DAD occurred in the PPC. We also assumed that the occurrence time of the PVC in tissues was the same as that of DADs in single cells. In addition, action potential duration (APD) was recorded as the time interval between the time of stimulus onset and 90% repolarization of the AP. Suprathreshold DAD was also defined as the ectopic beats, which can reach to the depolarization threshold required to trigger an AP.

### Model of intracellular pH restoration

Previous studies have indicated that the period of pH recovery might range from tens of seconds to a few minutes (Avkiran et al., 1996; Park et al., 1999; Inserte et al., 2009). Indirect data also has suggested that a delayed recovery of intracellular pH during reperfusion is involved in postconditioning protection (Maruki et al., 1993; Avkiran et al., 1996; Kin et al., 2004; Fujita et al., 2007; Inserte et al., 2008, 2009). Moreover, the change of intracellular pH during reperfusion was close to a linear variation (Inserte et al., 2009). To simulate a delayed pH recovery process, a linear function with different rates of the pH change was used to model pH restoration. Specifically, three different recovery processes were considered: (i) the fast recovery period of 0.1 min; (ii) the slow recovery period of 0.5 min; and (iii) the gradual recovery period of 4 min. In addition, multiple pH restoration protocols (P_X) with a recovery time of X min were modeled to evaluate the effect of pH restoration time on the probability of generating DADs. These protocols included P_0, P_0.2, P_0.5, P_1, P_2, P_3 and P_4. In each case, the number of DADs and the maximal DAD amplitude during the recovery period were quantified.

### Model of PVCs in a one-dimensional (1D) homogeneous cable

To investigate the cellular level conditions required for DADs to trigger a PVC at the multicellular tissue level, a 15-mm-long epicardial strand model consisting of 100 myocytes was constructed. The middle of the strand contained an acidotic region in which myocytes were set as DAD generating cells. Varying numbers of myocytes in the acidotic region (i.e., varying sizes of the acidotic region) were also considered. To evaluate the AP inducibility in the model, the SR calcium content (peak [Ca^2+^]_SR_) and the amplitude of cytoplasmic calcium transient (peak [Ca^2+^]_i_) associated with the DADs generating were measured. The DAD amplitude (DADA) was calculated as the difference between the resting potential and the maximum voltage of DADs. The voltage threshold of DADs was computed as the minimum DADA required to trigger an AP. The minimum number of cells (either generating subthreshold or suprathreshold DADs) required to develop a PVC in the strand was calculated. The effects of the SR calcium load and gap junction uncoupling on the minimal number of acidotic cells required to initiate a PVC were investigated to evaluate the sink-source relationship of cardiac tissues. As the SR calcium content gradually decreased after acidosis, peak [Ca^2+^]_SR_ was varied from the maximum [Ca^2+^]_SR_ at the end of acidosis to that at the end of post acidosis. In addition, increasing [Ca^2+^]_i_ may increase gap junction resistance (Noma and Tsuboi, 1987; Peracchia, 2004) and gap junctional uncoupling was also observed in ischaemic tissues. The extent of gap junctional uncoupling was modeled by decreasing the diffusion coefficient (*D*) (see Equation 1 in Numerical methods) from 100% (0.154 mm^2^/ms) to 80, 60, 40, and 20%.

### Model of unidirectional conduction block in a 1D transmural ventricular fiber

**Figure 1:**
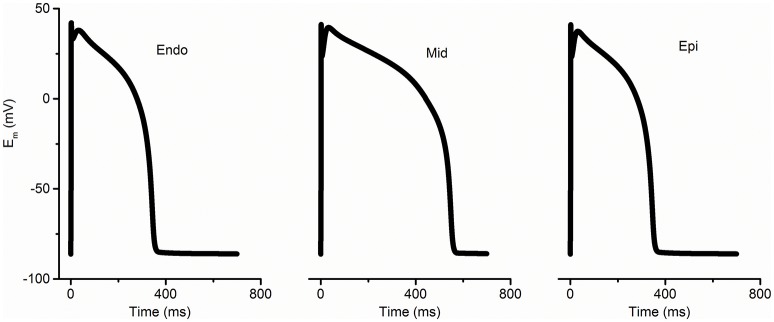
**Simulated membrane potentials (E_m_) with the modified TP 06 model (ten Tusscher and Panfilov, 2006) for endocardium (Endo), mid-myocardium (Mid), and epicardium (Epi) under normal conditions**.

To assess the role of repolarization dispersion in making tissue susceptible to unidirectional conduction block, a 15-mm-long transmural fiber model was developed. The fiber consisted of a 3.75-mm-long Endo region, a 5.25-mm-long Mid region and a 6-mm-long Epi region (APs for Endo cells, Mid cells and Epi cells under the basal condition are shown in Figure 1). The total length of the transmural cable was within the width range (~8–15 mm) of human ventricular wall (Drouin et al., 1995; Yan et al., 1998) and the proportions of each subdomain used in this study were consistent with those used in other studies (Zhang et al., 2008; Adeniran et al., 2012). The cellular uncoupling between the midmyocardium and epicardium was modeled with a 5-fold decrease in the diffusion coefficient at the epicardium-midmyocardium border, as previously suggested by Gima and Rudy (2002). To examine the electrical heterogeneity of the tissue, an excitation wave propagating from the endocardium to the epicardium was initiated by a stimulus (with an amplitude of −40 μA/cm^2^ and a duration of 1 ms), and the fiber repolarization time was measured as the latest repolarization time of cells in the fiber. TDR was quantified by computing the difference (the time interval between the earliest repolarization time and the latest repolarization time) in repolarization time along the fiber. The inducibility of unidirectional conduction block for a TDR value was quantified by computing the vulnerable window (VW), during which a PVC may evoke a unidirectional conduction wave. The unidirectional conduction block was created by applying a premature stimulus (for simulating an ectopic beat) to an epicardial region with a time delay after the previous stimulus. The increase in TDR (Levites et al., 1975) and the decrease in the current density of fast delayed rectifier potassium currents (IKr) (Jiang et al., 2000) were observed in ischaemic tissues. Moreover, the TDR may be mainly modulated by the midmyocardium, because APD prolongation in the midmyocardium is much greater than that in the endocardium or epicardium (Ueda et al., 2004). Therefore, the extent of TDR was varied by decreasing the conductance of IKr from 100% (Normal) to 80% (#1), 60% (#2), 40% (#3) and 20% (#4) in the midmyocardium to examine whether increased TDR promoted inducibility of unidirectional conduction block. Repolarization gradient was also calculated as repolarization time change rate per millimeter along the strand.

### Model of reentry in a 2D transmural ventricular sheet

To determine whether the DADs caused by the calcium overloaded SR at the cellular level can evolve into reentrant excitation waves at the tissue level, and to determine the effects of TDR and gap junctional uncoupling on the genesis of reentry, computer simulations were performed in a transmural ventricular tissue sheet (consisting of 100 × 500 grid points, with each point representing a cell). The 2D model was constructed by expanding the 1D model of a 15-mm-long transmural fiber into a 75-mm-wide sheet. In the model, the size of the local acidotic region was chosen to be 6 × 45 mm^2^ (corresponding to 40 × 300 DAD generating cells) such that it was large enough to trigger a PVC in the tissue. In simulations, a planar excitation wave, which was elicited by a stimulus applied to the end of the endocardium side, propagated toward the epicardial region. After the conditioning wave, a PVC was produced in the acidotic epicardial region. When the PVC occurred within VW of cardiac tissues, unidirectional conduction of the PVC-evoked excitation wave was observed, leading to the formation of a spiral wave. Dynamics of excitation waves under normal, increased TDR (#2) and the #2 TDR combined with slow conduction conditions were investigated.

### Numerical methods

The monodomain model in cardiac electrophysiology was used to describe the reaction-diffusion system in simulating cardiac dynamics (ten Tusscher and Panfilov, 2006). The governing equation is

(1) $$\frac{\partial E_{m}}{\partial t}=\frac{I}{C_{m}}+D\left\{\frac{\partial^{2}E_{m}}{\partial x^{2}}+\frac{\partial^{2}E_{m}}{\partial y^{2}}\right\}$$

where *C*_*m*_ = 1 μF/cm^2^ is the capacitance, *D* denotes the diffusion coefficient, *I* denotes the total transmembrane current and *V*_*m*_ is the membrane voltage. *D* was set to a constant value of 0.154 mm^2^/ms that gave a conduction velocity (CV) of 74.2 cm/s, which was similar to the CV of excitation waves in human heart (Taggart et al., 2000). Time step (Δ*t*) is 0.02 ms and space step (Δ*x* = Δ*y*) is 0.15 mm, which is close to the length of ventricular myocytes. The partial differential equations were solved by an explicit forward Euler approximation. Simulations were carried out on a 64 G memory with Intel core i703930K 64-bit CPU system. Efficient parallelization was implemented using GPU acceleration.

## Results

### Acidosis-induced electrophysiological changes at the subcellular level

**Figure 2:**
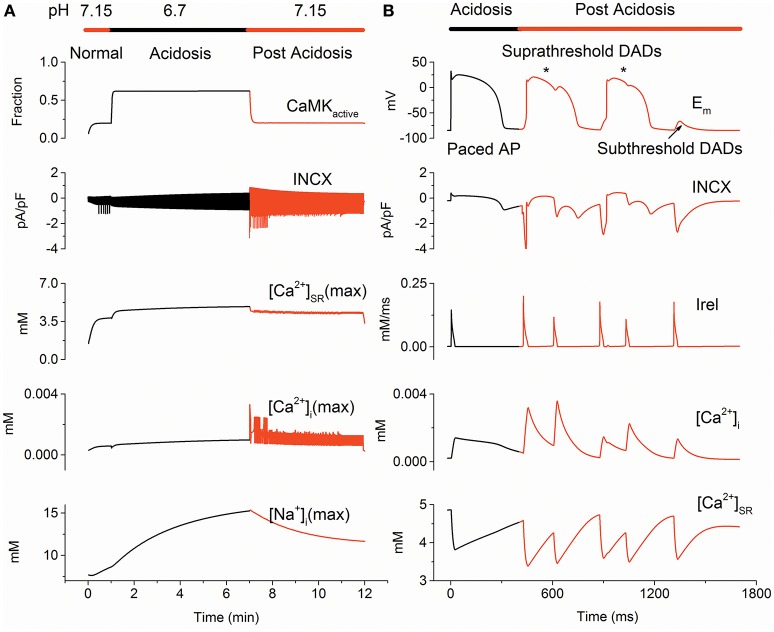
**The protocol of pH changes (PPC) predicted delayed afterdepolarizations (DADs) on returning to normal pH. (A)** Changes of CaMK_active_, INCX, [Ca^2+^]_SR_(max), [Ca^2+^]_i_(max) and [Na^+^]_i_(max) due to pH alterations in the PPC. **(B)** Time course of membrane potential (E_m_), INCX, Irel, [Ca^2+^]_i_ and [Ca^2+^]_SR_ during acidosis (Black) and post acidosis (Red). Suprathreshold DADs marked by an asterisk and subthreshold DADs marked with an arrow are shown. CaMK_active_, fraction of activated calcium/calmodulin-dependent protein kinase II; [Ca^2+^]_i_(max), maximum cytoplasmic calcium concentration; [Ca^2+^]_SR_(max), maximum sarcoplasmic reticulum calcium concentration; [Na^+^]_i_(max), maximum intracellular sodium concentration; INCX, the sodium-calcium exchanger current and Irel, sarcoplasmic reticulum calcium release flux.

Figure 2A illustrates altered calcium handling in the PPC during control, acidosis and post acidosis, and Supplementary Figure 4 is an enlarged view of changes in membrane potential (Em), [Na^+^]_i_, [Ca^2+^]_i_, [Ca^2+^]_SR_ and INCX at different times during the PPC. An arrhythmic pattern of phase-4 depolarization upon returning to normal pH was predicted (Figure 2B). The DAD was produced by the acidosis-induced increase in [Ca^2+^]_SR_, which, consequently, enhanced the SR calcium leak, accompanied by an inward INCX that depolarized the cell. Increased INCX contributed to the inward current responsible for membrane depolarization amplification, which may lead to suprathreshold DADs (Jie and Trayanova, 2010). As pH reached its normal value, [Ca^2+^]_SR_ decreased, leading to a gradual reduction in the INCX, which caused a gradual decrease in membrane depolarization, resulting in subthreshold DADs. These ectopic beats were characterized by membrane depolarization associated with abnormalities in INCX, Irel and [Ca^2+^]_i_ and [Ca^2+^]_SR_(Figure 2B).

To analyze altered calcium handling related to DADs generating, changes in CaMK_active_, INCX, [Ca^2+^]_i_(max), [Ca^2+^]_SR_(max) and [Na^+^]_i_(max) due to pH alterations were examined for the normal, acidosis and post-acidosis conditions. At the beginning of acidosis, there was a sharp decrease in [Ca^2+^]_i_(max), [Ca^2+^]_SR_(max) and INCX, then acidosis produced a gradual increase in [Na^+^]_i_(max), [Ca^2+^]_i_(max), [Ca^2+^]_SR_(max) and INCX, and restoration of the intracellular pH caused abnormal [Ca^2+^]_i_(max), [Ca^2+^]_SR_(max), INCX, leading to membrane depolarization (Figure 2A). In detail, acidosis increased [Ca^2+^]_SR_, which reached to a maximum value of 4.86 mM at the end of the acidosis period. After acidosis, the calcium overload SR augmented Irel, which led to the first suprathreshold DADs with an occurrence time of 420 ms. Then, [Ca^2+^]_SR_ decreased, consequently, resulting in a gradually decrease in the membrane depolarization, generating subthreshold DADs with an occurrence time of 1,315 ms (Figure 2B).

### Protection effects of delayed recovery of intracellular pH on the development of triggered activity at the cellular level

**Figure 3:**
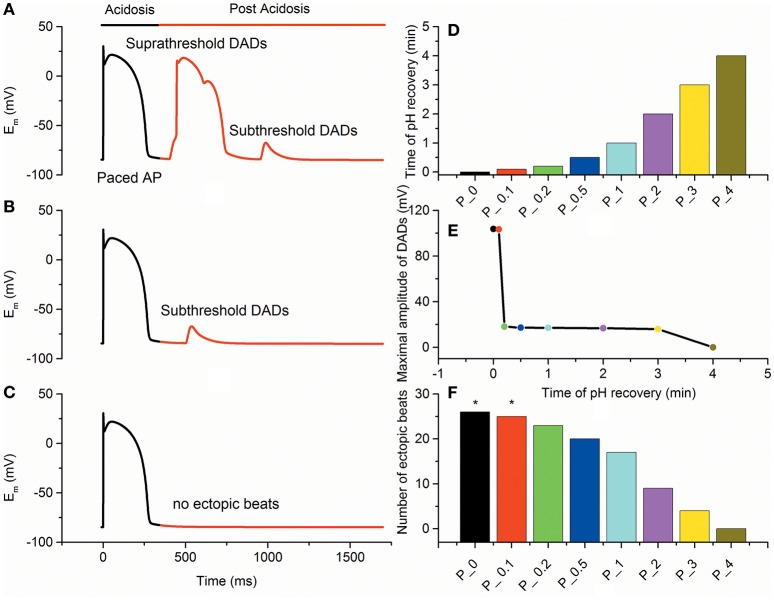
**Epicardial membrane potentials (E_m_) with different pH recovery protocols after acidosis. (A)** The fast pH recovery of 0.1 min triggered suprathreshold delayed afterdepolarizations (DADs). **(B)** The slow pH recovery of 0.5 min triggered subthreshold DADs. **(C)** The gradual pH recovery of 4 min suppressed the occurrence of DADs. **(D)** pH recovery time for different simulation protocols. Each bar represents the time of pH recovery for each protocol. **(E)** The maximal amplitude of DADs after acidosis gradually decreased with the recovery time of pH. **(F)** The number of ectopic beats measured under each condition. Suprathreshold DADs (marked by an asterisk) were triggered in P_0 and P_0.1.

To investigate the effects of the recovery time of intracellular pH on development of triggered activity in myocytes, three pH restoration protocols were used to examine the inducibility of triggered activity. With the fast pH recovery of 0.1 min, [Ca^2+^]_SR_ decreased suddenly, and the spontaneous SR calcium release increased quickly, leading to a suprathreshold DAD (Figure 3A). With the slow pH recovery of 0.5 min, [Ca^2+^]_SR_ decreased gradually, and the spontaneous SR calcium release was limited. As the pH restoration proceeded, subthreshold DAD was triggered (Figure 3B). With the gradual pH restoration of 4 min, delayed recovery of the intracellular pH attenuated the SR calcium load and suppressed the occurrence of DADs (Figure 3C).

Furthermore, multiple pH restoration protocols (P_X) with an X-min-long recovery time were used to assess the probability of generating DADs (Figure 3D). In simulations, no DAD was observed for slow pH recovery processes (t ≥ 4 min). However, when the pH recovered quickly (0.15 ≤ t < 4 min), the number of post-acidosis DADs increased, and subthreshold DADs were triggered. As the pH restoration became faster (0 ≤ t < 0.15 min), suprathreshold DADs were obtained (Figure 3E). In addition, the number of ectopic beats gradually decreased with the increase of pH restoration time (Figure 3F). With a time course of 0, 0.1, 0.2, 0.5, 1, 2, 3, and 4 min of pH recovery, the numbers of DADs during post acidosis were 26, 25, 23, 20, 17, 9, 4, and 0, respectively. Compared with the control condition (P_0), the incidences of DADs in P_0.1, P_0.2, P_0.5, P_1, P_2, P_3, and P_4 decreased by 4, 11, 23, 34, 65, 84, and 100%, respectively. In the simulation, at least 4 min of pH restoration was required to achieve sustained protection against DADs.

### Requirement for the PVCs development arising from DAD generating cells

**Figure 4:**
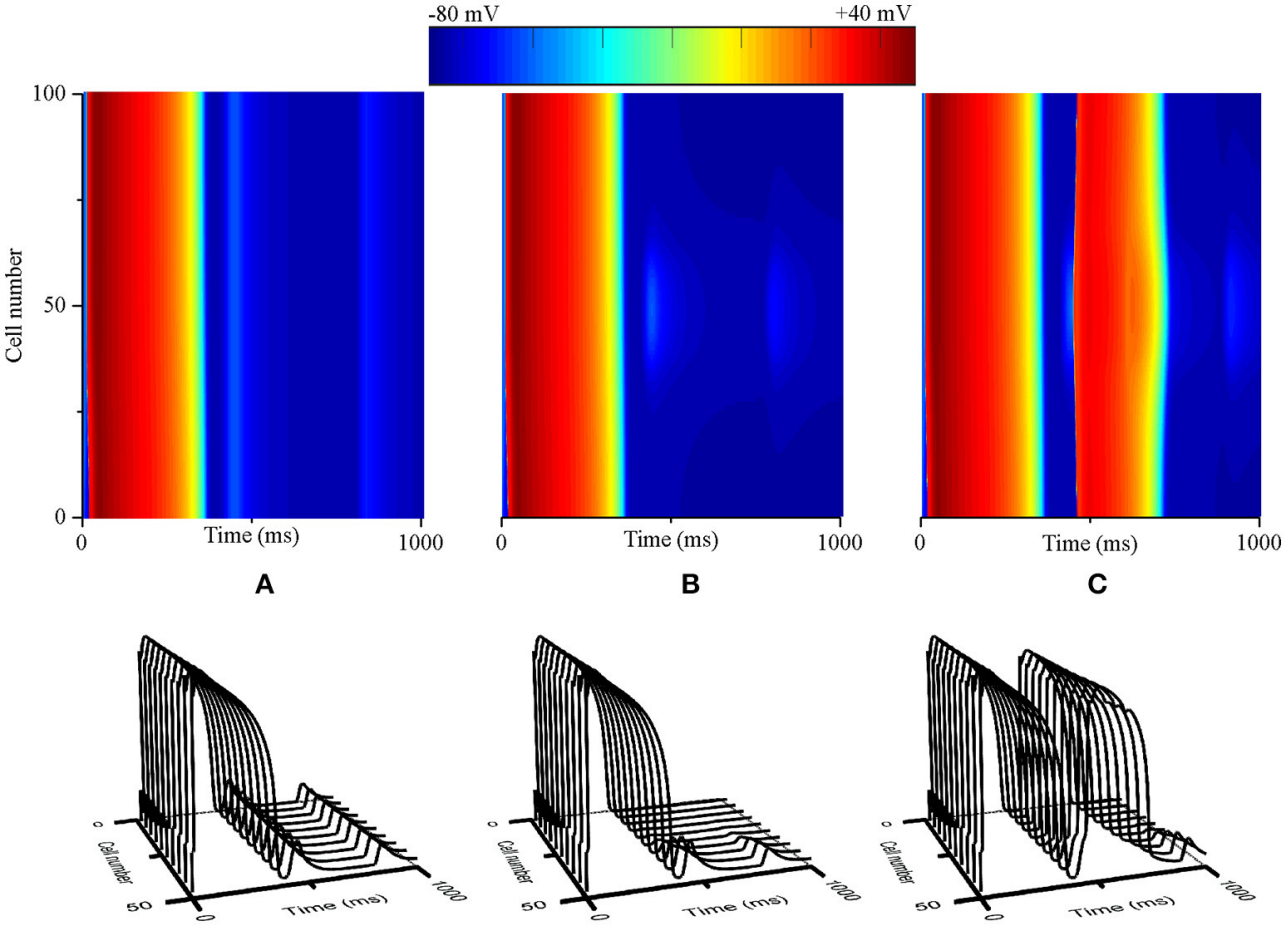
**Requirements for delayed afterdepolarizations (DADs) to produce a premature ventricular complex (PVC) in the 1D cable in which a central region of contiguous myocytes exhibiting DADs was surrounded by normal cells. (A)** No PVC produced due to subthreshold DADs in the central region. **(B)** No PVC generated, though cells in the central region exhibiting suprathreshold DAD but the length of the region is under a critical value (e.g., 24 cells). **(C)** A PVC generated as the length of the region exhibiting suprathreshold DAD is over a critical value (e.g., 25 cells).

The size of the acidotic region required to produce a PVC was examined in a 1D homogeneous strand. For the subthreshold DADs, although cells in the cable exhibited subthreshold DADs, the acidotic region failed to produce a PVC (Figure 4A). Similarly to subthreshold DADs, the acidotic region with 24 suprathreshold DAD cells could not overcome the sink-source mismatch to produce a propagating excitation wave (Figure 4B), while this region with 25 cells exhibiting suprathreshold DAD could do so (Figure 4C). Therefore, cells exhibiting suprathreshold DADs that act as a “source” of excitation could initiate excitation wave propagating in the surrounding normal cells (“sink”), generating a PVC at the fiber tissue level.

**Figure 5:**
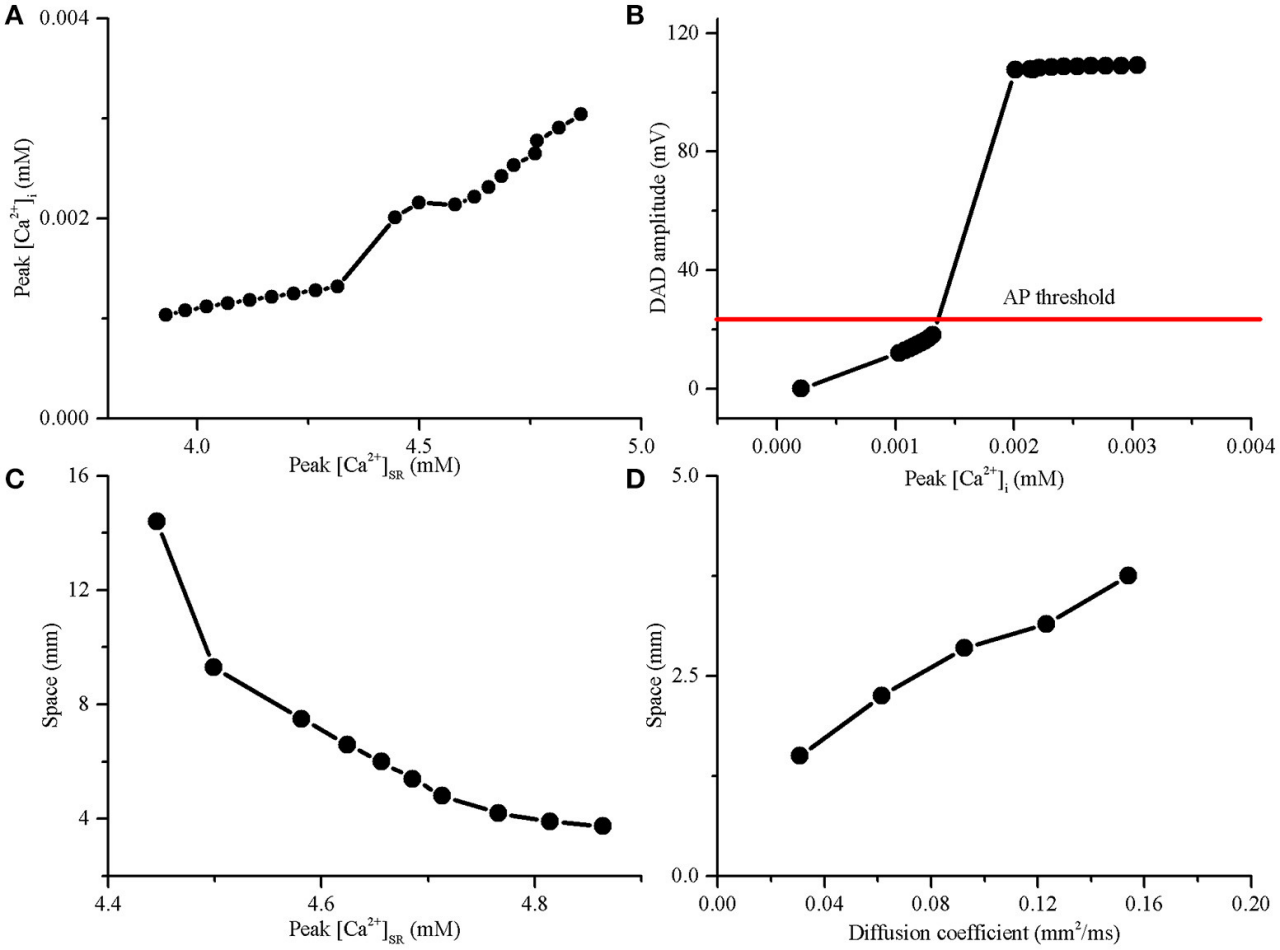
**The delayed afterdepolarization (DAD) amplitude required to trigger an action potential (AP) and the length of DAD generating region in a 15-mm-long 1D cable required to trigger a premature ventricular complex (PVC). (A)** Amplitude of cytoplasmic calcium transient (peak [Ca^2+^]_i_) increased with SR calcium content (peak [Ca^2+^]_SR_). **(B)** The DAD amplitude increased with the increase of peak [Ca^2+^]_i_ in a single myocyte. **(C)** The SR calcium overload significantly decreased the length of DAD generating region required to trigger a PVC. **(D)** Slow conduction by decreasing the diffusion coefficient can decrease the length of the DAD generating region for producing a PVC.

Analysis of subcellular SR function has shown that the DADA is generally [Ca^2+^]_SR_-dependent (Schlotthauer and Bers, 2000; Katra and Laurita, 2005). SR calcium overload increased the spontaneous calcium release, promoting membrane depolarization toward the threshold to trigger an AP, the precursor of PVC. The minimal value of peak [Ca^2+^]_SR_ contributing to the genesis of a suprathreshold DAD was 4.45 mM during post acidosis. The voltage threshold for inducing suprathreshold DADs was ~18.15 mV (from −84.15 to −66 mV) (Figures 5A,B). For the suprathreshold DAD case, the length of the acidotic region required to trigger a propagating excitation wave was 14.4 mm, corresponding to 96 myocytes. If the peak [Ca^2+^]_SR_ was augmented to increase the DADA further above the voltage threshold, the number of cells in the acidotic region decreased significantly (Figure 5C). The maximum [Ca^2+^]_SR_ during acidosis was 4.86 mM, and the corresponding DADA at this point was 109.1 mV; consequently, the minimal number of DAD generating cells required to elicit a propagating AP was 25 (3.75 mm). Acidosis-induced SR calcium overload may cause membrane depolarization after acidosis and consequently enhance the “source” of excitation required for the generation of PVCs.

To investigate the effect of gap junctional uncoupling on the development of PVCs, we performed simulations with the diffusion coefficient decreasing from 100 to 80, 60, 40, and 20%, which reduced CV from 74.2 to 65.8, 56.3, 45, and 30.1 cm/s, respectively. When the peak [Ca^2+^]_SR_ was 4.86 mM, the size of the acidotic region was significantly reduced with the decrease in diffusion coefficient. In detail, the length of the acidotic region required to trigger a PVC was reduced from 3.75 to 3.15, 2.85, 2.25, and 1.5 mm when the diffusion coefficient was decreased by 20, 40, 60, and 80%, respectively (Figure 5D). In the simulation, gap junctional uncoupling may increase tissue resistance, which could decrease the “sink” of the surrounding normal tissue, facilitating the PVCs generation.

Taken together, these simulations showed that acidosis-induced electrophysiological changes may increase the “source” of excitation and decrease the “sink” of cardiac tissues, resulting in increased susceptibility to PVCs during post acidosis.

### Pro-arrhythmic effects of altered electrical heterogeneity on inducibility of unidirectional conduction block at the fiber tissue level

**Figure 6:**
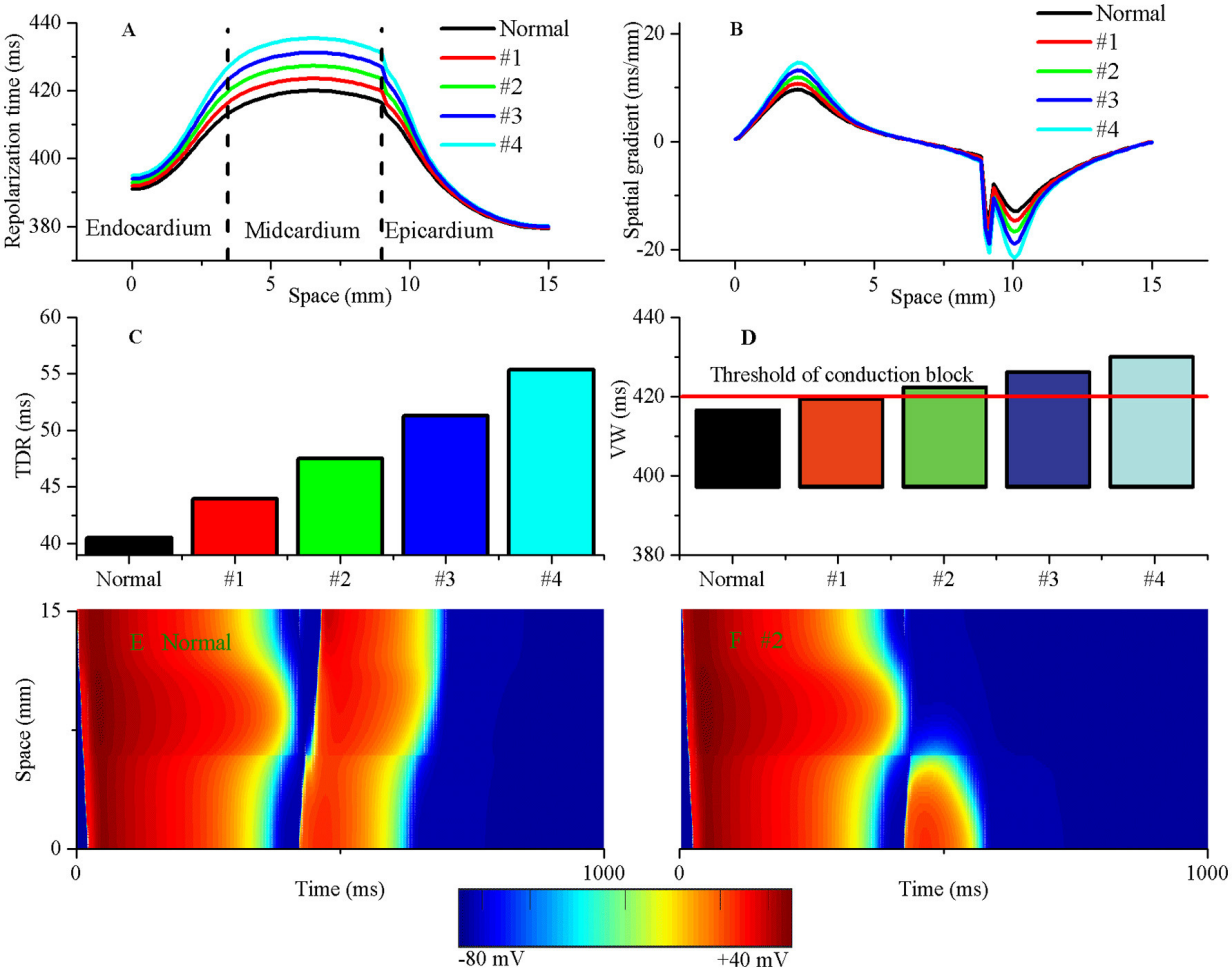
**The augmented transmural dispersion of repolarization (TDR) can increase tissue vulnerability to unidirectional conduction block**. The repolarization time **(A)**, spatial gradients of repolarization time **(B)**, TDR **(C)** and vulnerable window (VW) **(D)** are shown under normal, #1, #2, #3, and #4 conditions. The VW progressively grew when TDR was augmented. The threshold of unidirectional conduction block in post acidosis was equal to 420 ms when suprathreshold DADs occurred in single cells. **(E)** A premature ventricular complex (PVC) occurred without tissue's VW under the normal condition and produced a bidirectional conduction, whereas a unidirectional conduction formed when a PVC occurred within tissue's VW under the #2 condition **(F)**.

Excitation wave propagation in regionally ischaemic tissues indicated that the onset of reentry was associated with repolarization dispersion (Nash et al., 2003). To examine the effects of TDR on the initiation of reentry and determine the degree of TDR required to induce unidirectional conduction block in response to a PVC, a 1D transmural fiber containing an acidotic region large enough to produce a PVC was used to perform simulations. A stimulus was applied to the first 3 cells at the endocardial (Endo) end, and the ability of the initiated wave to propagate to the epicardial (Epi) region was studied under five conditions (including Normal, #1, #2, #3, and #4). The repolarization time increased from 420 ms (Normal) to 423.5 ms (#1), 427.3 ms (#2), 431.2 ms (#3), and 435.4 ms (#4) (Figure 6A). The TDR due to the electrophysiological heterogeneity of cell types across the ventricular wall was also augmented from 40.52 ms (Normal) to 43.93 ms (#1), 47.52 ms (#2), 51.3 ms(#3), and 55.34 ms (#4), respectively (Figure 6C). Most importantly, cellular uncoupling between the subepicardium and midmyocardium resulted in an abrupt increase in the repolarization gradient from 14.6 ms/mm (Normal) to 16.0 ms/mm (#1), 17.5 ms/mm (#2), 19.0 ms/mm (#3), and 20.6 ms/mm (#4), respectively (Figure 6B). The augmented TDR increased the VW (Figure 6D) and produced steep spatial gradients of repolarization that may be responsible for unidirectional conduction block (Figure 6F). In detail, for the normal TDR, the repolarization gradient between the subepicardium and midmyocardium in response to the local PVC failed to cause a unidirectional conduction (Figure 6E), because the upper limit of the VW in the normal settings was below the occurrence time of the PVCs induced in the post-acidosis period (420 ms, threshold of unidirectional conduction block) (Figure 6D). If the TDR was progressively increased, as might occur physiologically during acidosis (Jie et al., 2010), unidirectional conduction block generated when the TDR was above the critical threshold (>43.93 ms). In the #2 case, the tissue surrounding the local acidotic region partly recovered for re-excitation, and an excitation wave induced by the post-acidosis PVC could propagate to the recovered tissue (the subepicardium) and be blocked by the unrecovered tissue (the midmyocardium), so unidirectional conduction was obtained (Figure 6F). The findings implied that increased TDR in response to a PVC which can arising from an acidotic region may lead to unidirectional conduction block favoring the development of reentry.

### The reentry evaluation from PVCs at the tissue sheet level

**Figure 7:**
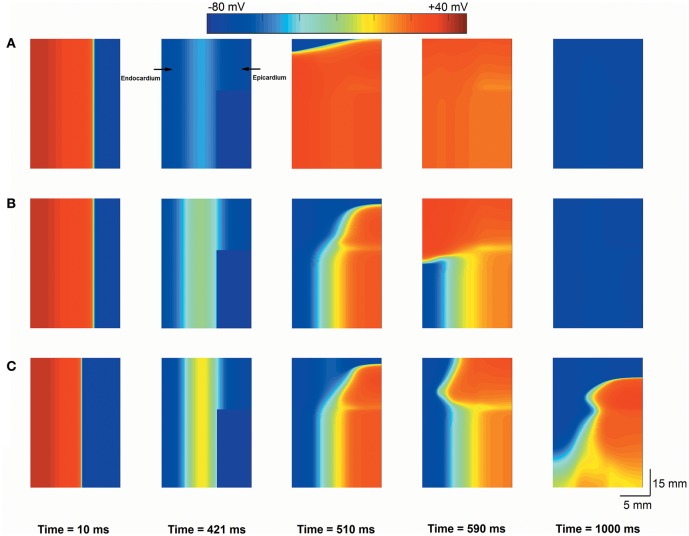
**Snapshots of excitation waves in a 2D transmural ventricular tissue**. Under the normal transmural dispersion of repolarization (TDR) **(A)**, #2 TDR **(B)** and #2 TDR combined with slow conduction **(C)** conditions, a planar conditioning wave generated at the end of the endocardium and propagated to the epicardium (Time = 10 ms), and then a premature ventricular complex (PVC) due to the acidotic region occurred at Time = 420 ms. Bidirectional conduction **(A)** and unidirectional conduction block **(B,C)** were observed (Time = 590 ms), but a spiral wave self-terminated under the #2 TDR condition **(B)** and maintained in #2 TDR combined with slow conduction settings **(C)** (Time = 1,000 ms).

To determine whether interactions between triggered activity and local repolarization dispersion produce reentry, further simulations using a 2D transmural tissue model with an acidotic region were conducted to examine the initiation of reentry caused by PVCs and altered electrical heterogeneity. For the normal condition, a planar conditioning wave was initiated by a conditioning stimulus that propagated from the endocardium to the epicardium (Figure 7A, Time = 10 ms), and then the acidotic region generated a PVC which occurred at 420 ms and propagated to the surrounding tissue (Figure 7A, Time = 421 ms). However, the PVC produced bidirectional conduction (Figure 7A, Time = 510 ms) and disappeared at 840 ms, because the occurrence time of the PVC was out of VW. When the TDR was increased, the PVC occurred at 420 ms which is within VW under cases #2, #3, and #4 (Figure 6D). Thus, in the #2 case, the 2D tissue generated unidirectional conduction block in response to the PVC and promoted the initiation of reentry (Figure 7B, Time = 590 ms), but the spiral wave spontaneously terminated within 930 ms. The reason for this result may be that the tissue size could not support the spiral wave due to the large wavelength (WL) (WL = APD × CV). If the WL was shortened by slowing the conduction, i.e., as observed in ischaemic patients with gap junctional uncoupling, the spiral wave was self-maintained throughout the period of simulation in the #2 case with the reduction in *D* from 0.154 mm^2^/ms to 0.0616 mm^2^/ms, which corresponded to a decrease in CV from 74.2 to 45 cm/s (Figure 7C). The persistent reentrant waves can be explained by the short WL, resulting in a reduction in the size of the substrate required to facilitate and maintain reentry. Video files showing re-entry in 2D models are included in Supplementary Video 1.

The re-entry initiation is not only facilitated by the transmural heterogeneity, but also promoted by the tissue anisotropy. An anisotropic 2D ventricular tissue sheet with an acidotic island (Supplementary Figure 2) was also modeled to explain the re-entry initiation. The effect of pH restoration time on the inducibility of reentrant arrhythmias was investigated. Dynamics of excitation waves under the suprathreshold DADs (Supplementary Figure 3A), subthreshold DADs (Supplementary Figure 3B) and normal (Supplementary Figure 3C) conditions were investigated. Details can be found in Supplementary Material. The suprathreshold DADs evolved into a PVC, and finally produced a figure-of-eight reentry in the anisotropic tissue (Supplementary Figure 3A).

## Discussion

### Summary of major findings

The present study was an attempt to provide mechanistic insight into the complex electrophysiological effect of acute acidosis on the evolution of reentrant arrhythmia at the tissue level. It was a comprehensive framework for understanding the mechanisms responsible for postacidotic arrhythmias originated from microscopic electrophysiological alterations. In this work, acidosis was assumed to affect calcium handling at the subcellular level, kinetic properties of ion channels at the cellular level, and electrical heterogeneity and gap junctional coupling at the tissue level. Under these circumstances, simulation results indicated that reentry can be spontaneously initiated in cardiac tissues during post acidosis and that the initiation of reentry was affected by recovery time of intracellular pH, the extent of SR calcium load, the size of the acidotic zone, the extent of repolarization dispersion and the degree of gap junction uncoupling. To unravel the mechanisms underlying arrhythmogenesis after acidosis, effect of pH restoration time on the inducibility of DADs, effect of SR calcium overload and gap junction uncoupling on the source-sink relationship required to the PVCs producing and effect of altered repolarization dispersion on the development of unidirectional conduction block necessary to the genesis of reentry were investigated. It was shown that acute pH restoration during post acidosis modified the balance of ion flow inside and outside the ventricular cell, resulting in DADs. These DADs may increase the “source” of excitation, while gap junction uncoupling can decrease the “sink” of cardiac tissue, both of which mediated the source-sink balance that can constitute the requirements for PVC generation. Synchronized DADs can overcome the source-sink mismatch and produce a PVC in ventricular tissues. The conduction of a PVC can be unidirectionally blocked and form a spiral wave if the PVC occurs within the VW of the tissue. And acidotic tissue may increase electrophysiological heterogeneity and consequently alter repolarization dispersion and elevate the upper limit of VW, increasing the susceptibility to re-entrant arrhythmias. These mechanisms are discussed in detail in the sections to follow.

### Mechanistic insights

SR calcium overload has been linked to arrhythmias after acidosis. Experimental and computational studies have shown that ectopic activity occur after acidosis when the SR becomes calcium-overloaded for an increase in CaMKII phosphorylation of PLN (Mattiazzi et al., 2007; Said et al., 2008; Pedersen et al., 2009; Lascano et al., 2013). DAD-induced triggered activity was prevented by decreasing the SR calcium content upon application of a CaMKII inhibitor. The DADA, which depended on the extent of the SR calcium load, may determine the inducibility of arrhythmias. Since suprathreshold DADs can initiate APs for propagation to adjacent cells, the results support the notion of a major role of SR calcium overload in the onset of ectopic activity. Recent experimental data indicated that a rabbit heart with focal arrhythmia showed a consistent increase in SR calcium content (Schlotthauer and Bers, 2000) and suprathreshold DADs after acidosis were observed in rat-isolated epicardial myocytes (Said et al., 2008). In agreement with previous findings, the simulation results showed that [Na^+^]_i_, [Ca^2+^]_SR_, and [Ca^2+^]_i_ gradually accumulated during acidosis. An increase in diastolic [Ca^2+^]_i_ caused by SR calcium leak was also predicted during post acidosis. The SR calcium leak due to the SR calcium load greatly augmented the incidence of calcium-induced transient depolariztheation after acidosis. Importantly, the DADA can reach the threshold to trigger an AP, thus increasing the likelihood of having PVCs.

It is more likely to produce PVCs during post acidosis at the beginning than at the end. The simulation results support the common perception that the SR calcium load resulting from acidosis is pro-arrhythmic. Synchronized SR calcium release due to SR calcium load can generate a PVC after acidosis. The propensity for PVCs to occur is consistent with experimental observations (Fujiwara et al., 2008). Local β-adrenergic receptor stimulation caused spatiotemporal synchronization of the SR calcium overload and DADs, and these synchronized DADs overcame the sink impedance to trigger focal arrhythmia in the rabbit heart (Myles et al., 2012). Although PVC inducibility was related to area of the DAD generating region, the difference in the same area of acidotic regions did not account for the high arrhythmia propensity at beginning of the post-acidosis period. The mechanisms underlying the increased arrhythmogenic potentials upon returning to normal pH were not fully characterized. The simulation results suggest a mechanism by which SR calcium load after acidosis may increase the incidence of arrhythmias. It is well established that the area of DAD generating region is proportional to the amount of calcium in the SR (Xie et al., 2010; Myles et al., 2012). A larger amount of SR calcium might lead to an increase in the DADA, resulting in a “source” of excitation, thus significantly decreasing the area of acidotic regions required to induce a PVC. Indeed, it was shown that peak [Ca^2+^]_SR_ reached the maximum at the end of acidosis. The maximum [Ca^2+^]_SR_ caused the SR calcium release, leading to the maximum DADA. Thus, the number of DAD generating myocytes required to initiate a PVC was the minimum upon returning to normal pH, and consequently the maximal propensity to PVCs.

Delayed recovery of intracellular acidosis during post-acidosis can prevent the triggering of ventricular arrhythmias (Avkiran et al., 1996). It was shown that the prolongation of intracellular acidosis could suppress the occurrence of DADs. The results further suggested that postconditioning protection may depend on the prolongation of pH recovery during reperfusion and at least 4 min of pH restoration was required to achieve sustained protection against DADs. The minimum time for pH restoration required for this protection is within the time range of rat hearts (2–4 min) (Avkiran et al., 1996; Fujita et al., 2007). Previous studies have shown that, acidic reperfusion can significantly suppress the incidence of ventricular fibrillation in regionally ischaemic isolated rat hearts and that the protective mechanism may involve enhanced recovery of sodium-potassium-ATPase activity as well as inhibition of sodium influx (Avkiran et al., 1996); this assertion was confirmed in a previous simulation study that suggested increased intracellular sodium was a critical determinant of SR reloading and hence DAD maintenance (Lascano et al., 2013). In addition, postconditioning protection may contribute to prolongation of intracellular acidosis during reperfusion. At least 2 min of acidic reperfusion was required to achieve sustained protection against VF (Avkiran et al., 1996), and a further delay in pH recovery (>3.5) could improve functional recovery and reduce infarct size (Inserte et al., 2009). Hence, the results may be used to explain the postconditioning protection during reperfusion.

Repolarization dispersion provided a key connection between PVCs and VT in cardiac tissues. It was shown that an increase in tissue electrophysiological heterogeneity promoted the onset of reentry in the transmural ventricular tissue with an acidotic region; the acidotic region can generate a PVC. The results support the previous notion that an increase in repolarization dispersion plays an important role in the genesis of reentry (Wu and Zipes, 2001, 2002). It is possible that unidirectional conduction block occurs in the presence of a PVC, since the repolarization dispersion may increase the tissue VW and a PVC arising from an acidotic region is easily triggered within the VW ]the first PVC occurred at 420 ms, which is more than, but fairly close to, the maximum time in the rabbit heart (from 300 to 400 ms)] (Myles et al., 2012). Previous studies have shown that PVCs mainly occur in the epicardium and that the TDR is significantly increased during acidosis (Said et al., 2008). In addition, unidirectional conduction block due to cellular uncoupling between midmyocardium and epicardium was also observed (de Groot et al., 2001). Hence, the transmural ventricular tissue with acidotic regions likely increased the incidence of unidirectional conduction block, thus facilitating the initiation of reentry, which may be used to explain the occurrence of VT observed in patients. These findings were supported by experimental studies that showed the decline in TDR prevented the generation of VT (Dhalla et al., 2009).

We also found that slow conduction, which may occur under conditions of gap junctional uncoupling, increased susceptibility to post-acidosis arrhythmias. These findings were supported by a previous study which has suggested an extreme reduction in the number of gap junction channels is arrhythmogenic during ischaemia-reperfusion (Sánchez et al., 2011). On the one hand, the number of contiguous myocytes exhibiting suprathreshold DADs for producing a propagating PVC was significantly reduced in gap junctional uncoupling settings. This effect can be attributed to a reduction in the sink size of the surrounding tissue, so the minimal source of excitation required to trigger a PVC decreased. On the other hand, the size of transmural ventricular tissues necessary to produce persistent spiral waves was greatly decreased in gap junctional uncoupling settings. Slow conduction is thought to lead to a decrease in WL. The results showed that spiral waves were self-maintained under slow conduction conditions. Therefore, our findings support the assertion that gap junctional uncoupling may increase the risk for arrhythmias.

### Relevance to previous simulation studies

Crampin et al. developed a dynamical model of pH regulation and used the Luo-Rudy-dynamic (LRd) model to investigate possible roles of acidosis on key ionic species. Calcium and sodium loading were predicted in their simulations. They suggested that the most significant effects of acidosis were elevated [Na^+^]_i_, inhibition of NCX, and the direct interaction of protons with the contractile machinery (Crampin and Smith, 2006).

Crampin et al. also carried out a mathematical study on acidosis to determine the effects of changes in pH on the membrane potential and calcium handling. They observed increased [Ca^2+^]_i_ at both peak and resting levels and AP shortening. They suggested that the rise in [Na^+^]_i_ mediated changes in [Ca^2+^]_i_ (Crampin et al., 2006).

Lascano et al. modified a human myocyte model consisting of CaMKII effects on ion flows and contractile constants, and investigated the molecular mechanisms underlying the triggered. They observed SR calcium loading and post-acidotic DADs upon returning to normal pH. They concluded that DADs in single cells depended on CaMKII effect on L-type calcium channel and SERCA2a (Lascano et al., 2013).

We have developed a human ventricular acidotic model consisting of pH and CaMKII regulations, and analyzed the functional influence of acidosis on cardiac electrical activity and ECGs. We observed heterogeneous APD abbreviation and a PVC in the simulated ECG waveform (Liu et al., 2016).

Our simulation results are in agreement with and extend the findings of these previous studies, adding to the possibilities that acidosis-induced electrical alterations may mediate tissue's source-sink interactions and consequently underlie the evolution from acidosis-induced DADs at the cellular level to PVCs at the tissue level, that acidosis-induced electrical heterogeneity can increase spatial gradients of repolarization and facilitate the evolution from PVCs into re-entry at the sheet tissue level, and that prolonged transient acidosis during the early reperfusion phase may underlie the protective mechanism of ventricular arrhythmogenesis.

### Significance of the study

It is well known that the heart becomes acid in a number of pathological conditions, most dramatically during ischaemia-reperfusion. It has been suggested that the development of arrhythmias during reperfusion is due to the associated acidosis (Orchard et al., 1987; Orchard and Cingolani, 1994; Avkiran et al., 1996; Said et al., 2008; Kapur et al., 2009; Nagai et al., 2010; Niwano and Tojo, 2010; Lascano et al., 2013). However, it is difficult to identify mechanisms underlying these effects of acidosis because the substrates of ventricular arrhythmias change too rapidly to be observed during IRI in patients. Further studies have also shown that electrical alterations, which provide a potential mechanistic link between acidosis and ventricular arrhythmias, occur during ischaemia-reperfusion (Pinto and Boyden, 1999). Electrical changes (e.g., calcium handling and afterdepolarizations) of ventricular myocyctes have been observed in a number of animal models of ischaemia-reperfusion (Orchard et al., 1987; Avkiran et al., 1996; Wu and Zipes, 2001; Said et al., 2008; Dhalla et al., 2009; Kapur et al., 2009), as well as in human ischaemia-reperfusion (Adams and Pelter, 2002). Structural alterations (including gap junctional uncoupling) of ventricular tissues were also observed in isolated mouse hearts of ischemia-reperfusion and in patients with persistent VF (Luqman et al., 2007). Thus, this study may provide insight into the mechanisms underlying the ischaemia-reperfusion induced ventricular arrhythmias.

### Clinical implications

The prolonged transient acidosis has important clinical implications. The heart was protected against acute myocardial infarction by interrupting myocardial reperfusion with several short-lived episodes of myocardial ischaemia, a phenomenon termed “ischaemic postconditioning (IPost).” IPost was found to confer a myriad of protective effects, including reduced levels of myocardial oedema, oxidative stress, and polymorphonuclear neutrophil accumulation, as well as preserved endothelial function (Hausenloy and Yellon, 2016). In addition, IPost is associated with the protection against ventricular arrhythmogenesis (Avkiran et al., 1996), perhaps related to low inducibility of triggered activity via prolonged transient acidosis during the early reperfusion phase.

### Limitations

The limitations (e.g., the model lacks the ability to simulate calcium waves and contractility) of the original TP06 model have been discussed elsewhere (ten Tusscher and Panfilov, 2006; Zhang et al., 2008; ten Tusscher et al., 2009). Here we explain several limitations specific to the work. Firstly, the geometry structure of tissue sheet used in simulations is idealized rather than realistic. Although the model geometry does not represent that of the realistic ventricular slice, the use of an idealized geometry was necessary to enable elucidation of the mechanism of arrhythmogenesis which was caused by electrical heterogeneity rather than geometrical structure. Secondly, SR calcium release randomly occurred in single cells and the exact time of PVCs in ventricular tissues was not directly measured, so this study assumed that the concurrence time of PVCs was the same as that of DADs in single cell. It may affect reentry initiation, however, the mechanisms uncovered here will remain valid. Thirdly, our model does not account for the spatial gradient and shape of the local acidotic region, which would cause repolarization dispersion in the acidotic region. However, the study focused on the effect of electrical intrinsic heterogeneity on arrhythmogenesis in post acidosis and this design can support the uncovered mechanisms by eliminating the influence of the spatial gradient from the acidotic region. Fourthly, our model simulated the increase in TDR by prolonging repolarization of the midmyocardium with decreased the conductance of IKr,but many ion currents changed in ischaemic tissues. Special attention should be paid while trying to conduct data analysis. Fifthly, due to the lack of a precise measurement of gap junction uncoupling, the relative efficacy among the decrease in the diffusion coefficient might be model specific and further refinement of these models is required. Sixthly, the kinetics of pH recovery were modeled with a linear function which was not realistic, but protection mechanisms upon prolongation of pH recovery will remain valid. Seventhly, even though acidosis is likely to occur during ischemia/reperfusion, electrical alterations considered in our study may result from other effects of ischemia and special caution should be paid in using these simulated results. Eighthly, early afterdepolarizations (EADs) were also observed, but the ionic mechanism underlying EADs during reperfusion is complex and role of EADs in post-acidosis arrhythmias remains incompletely understood, which warrants further study in future. Despite these limitations, the study may provide detailed mechanisms underlying the evolution of VT from acute regional acidosis.

## Conclusion

The multi-scale cardiac modeling provides a framework to explain the evolution of postacidotic arrhythmias. Through multi-scale acidotic models, the relationship between acidosis-induced electrophysiological changes and ventricular arrhythmogenesis was built. The simulation results suggest that although SR calcium overload is a well-known ionic mechanism of triggered activity, source-sink interactions and electrical heterogeneity are critical determinants of the emergence of post-acidosis arrhythmias.

## Author contributions

JB and KW conceived and designed the experiments; JB and RY performed the simulations, prepared figures and analyzed the results; JB, RY, KW, and HZ drafted and edited the manuscript. All authors reviewed the final version of the manuscript.

## Funding

This work was supported by project grants from the National Natural Science Foundation of China (Contract No: 61571165 and 61572152).

### Conflict of interest statement

The authors declare that the research was conducted in the absence of any commercial or financial relationships that could be construed as a potential conflict of interest.
