# Supplementary material for: Mechanisms Underlying the Emergence of Post-acidosis Arrhythmia at the Tissue Level: A Theoretical Study
Source: Front Physiol. 2017 Mar 30;8:195. doi: 10.3389/fphys.2017.00195 (PMC5371659; doi:10.3389/fphys.2017.00195)
Supplement: Supplementary file 2 [file DataSheet1.DOCX]

Supplementary Material

Mechanisms underlying the emergence of post-acidosis arrhythmia at the tissue level: A theoretical study

Jieyun Bai, Renli Yin, Kuanquan Wang*, Henggui Zhang*

*** Correspondence:** Kuanquan Wang: wangkq@hit.edu.cn, [wangkq@ieee.org](mailto:wangkq@ieee.org)

Henggui Zhang:henggui.zhang@manchester.ac.uk

# A human ventricular cell model with CaMKII activation and pH regulation

## Sarcoplasmic reticulum (SR) calcium release model

SR calcium release (I_rel_) from the SR ryanodine receptor (RyR2) was modelled with the combination of both calcium-induced-calcium release and SR calcium leak. The I_rel_ model was equivalent to that used in the Lascano et al. human model(Lascano et al., 2013)

$$\boldsymbol{V}_{\boldsymbol{rel}}\boldsymbol{=0.102}\boldsymbol{ms}^{\boldsymbol{-1}}$$

$$\boldsymbol{V}_{\boldsymbol{sp}}\boldsymbol{=0.00036}\boldsymbol{ms}^{\boldsymbol{-1}}$$

$$\boldsymbol{I}_{\boldsymbol{rel}}\boldsymbol{=}\left( \boldsymbol{V}_{\boldsymbol{rel}}\boldsymbol{\cdot O+}\boldsymbol{V}_{\boldsymbol{sp}}\boldsymbol{\cdot R} \right)\boldsymbol{\cdot}\left( \left[ \boldsymbol{Ca}^{\boldsymbol{2+}} \right]_{\boldsymbol{SR}}\boldsymbol{-}\left[ \boldsymbol{Ca}^{\boldsymbol{2+}} \right]_{\boldsymbol{SS}} \right)$$

where V_rel_ denotes a rate constant of SR calcium release, V_sp_ denotes a rate constant of SR calcium leak, O denotes the open state of RyR2, R denotes the close state of RyR2, [Ca^2+^]_SR_ denotes the concentration of calcium ion in sub-cellular compartment SR (mM) and [Ca^2+^]_SS_ denotes the concentration of calcium ion in sub-cellular compartment dyadic cleft (SS) (mM). Equations about O, R, [Ca^2+^]_SR_ and [Ca^2+^]_SS_ were equivalent to that used in TP06 model(ten Tusscher and Panfilov, 2006).

## Calcium/Calmodulin-dependent protein kinase II (CaMKII) model

The CaMKII model was equivalent to that used in the Hund-Decker-Rudy dog model(Decker et al., 2009; O'Hara et al., 2011). We assumed that CaMKII kinetics are similar in human and dog, in the absence of human ventricle specific measurements.

$$\boldsymbol{CaMK}_{\boldsymbol{0}}\boldsymbol{=0.05}$$

$$\boldsymbol{\beta}_{\boldsymbol{CaMK}}\boldsymbol{=0.00068}\boldsymbol{ms}^{\boldsymbol{-1}}$$

$$\boldsymbol{K}_{\boldsymbol{mCaM}}\boldsymbol{=0.0015 mM}$$

$$\boldsymbol{CaMK}_{\boldsymbol{bound}}\boldsymbol{=}\boldsymbol{CaMK}_{\boldsymbol{0}}\boldsymbol{\cdot}\frac{\boldsymbol{1-}\boldsymbol{CaMK}_{\boldsymbol{trap}}}{\boldsymbol{1+}\frac{\boldsymbol{K}_{\boldsymbol{mCaM}}}{\left[ \boldsymbol{Ca}^{\boldsymbol{2+}} \right]_{\boldsymbol{SS}}}}$$

$$\frac{\boldsymbol{d}\boldsymbol{CaMK}_{\boldsymbol{trap}}}{\boldsymbol{dt}}\boldsymbol{=}\boldsymbol{\alpha}_{\boldsymbol{CaMK}}\boldsymbol{\cdot}\boldsymbol{CaMK}_{\boldsymbol{bound}}\boldsymbol{\cdot}\left( \boldsymbol{CaMK}_{\boldsymbol{bound}}\boldsymbol{+}\boldsymbol{CaMK}_{\boldsymbol{trap}} \right)\boldsymbol{-}\boldsymbol{\beta}_{\boldsymbol{CaMK}}\boldsymbol{\cdot}\boldsymbol{CaMK}_{\boldsymbol{trap}}$$

$${{CaMK}_{active}=CaMK}_{bound}+{CaMK}_{trap}$$

where CaMK denotes Calcium/Calmodulin-dependent protein kinase, CaMK_0_ denotes fraction of active CaMKII binding sites at equilibrium, β_CaMK_ denotes dephosphorylation rates of CaMKII, K_mCaM_ denotes half-saturation concentration of calmodulin, CaMK_bound_ denotes fraction of CaMKII binding sites bound to calcium/calmodulin, CaMK_trap_ denotes fraction of autonomous CaMKII binding sites with trapped calmodulin, CaMK_active_ denotes fraction of active CaMKII binding sites and α_CaMK_ denotes phosphorylation rates of CaMKII (ms^-1^).

## Ion currents regulated by CaMKII activation and intercellular pH

The total current (I) includes the basal current (I_b_), the increased current fraction produced by CaMKII activation(O'Hara et al., 2011) and the augmented current fraction created by pH regulation(Crampin and Smith, 2006). fe is CaMKII factor and fh is pH factor. Incorporating fh and fe into I, results in

$$\boldsymbol{fe=1+}\frac{\boldsymbol{IF}_{\boldsymbol{CaMK}}}{\boldsymbol{1+}\frac{\boldsymbol{K}_{\boldsymbol{mCaMK}}}{\boldsymbol{CaMK}_{\boldsymbol{active}}}}$$

$$\boldsymbol{fh=}\frac{\boldsymbol{f}_{\boldsymbol{0}}}{\boldsymbol{1+}\boldsymbol{10}^{\boldsymbol{n}\left( \boldsymbol{-pH+PK} \right)}}$$

$$\boldsymbol{I=fh\cdot fe\cdot}\boldsymbol{I}_{\boldsymbol{b}}$$

that takes into account both CaMK_active_ and pH effects on the corresponding ion currents.

For fe calculation, mean IF_CaMK_ was 0.25 for L-type calcium current (I_CaL_), 0.05 for I_rel_, 0.45 for SR calcium ATPase2a flux (I_up_), 0.08 for transient out current (I_to_), and 0.2 for late sodium current (I_NaL_). To maintain the TP06 model structure, α_CaMK_ was set 0.0135 ms^-1^ and fh was set 1 at pH=7.15. For pH=6.7, f_0_, n and PK were assigned different values on each pH target(Lascano et al., 2013) (**Supplementary Table 1**), and α_CaMK_ was set 0.035 ms^-1^ (model fit from the reference(Lascano et al., 2013)).

**Supplementary Table 1**. fh parameters f_0_, n and PK for pH (6.7) targets.

| pH target | f0 | n | PK | fh |
| --- | --- | --- | --- | --- |
| I_CaL_ | 1.11 | 1.53 | 6.52 | 0.72 |
| I_rel_ | 1.11 | 1.87 | 6.64 | 0.627 |
| I_up_ | 3.71 | 1.14 | 7.53 | 0.377 |
| I_NCX_ | 2.65 | 0.99 | 7.37 | 0.472 |
| I_NaK_ | 1.43 | -0.86 | 6.72 | 0.7 |
| I_K1_ | 1.43 | -1.41 | 6.89 | 0.5 |
| I_to_ | 1.43 | -0.86 | 6.72 | 0.7 |

Note: I_CaL_ denotes L-type calcium current, I_rel_ denotes SR calcium release flux, I_up_ denotes SR calcium ATPase2a flux, I_NCX_ denotes sodium-calcium exchanger current, I_NaK_ denotes sodium-potassium pump current, I_K1_denotes inward rectifier potassium current and I_to_ denotes transient out current.

## Model behavior

To test that pH modifications was responsible for variable changes, their response at pH=7.15 and pH= 6.7 were simulated. After stabilization, I_CaL_, I_NCX_, I_rel_, I_up_, I_to_, I_K1_, I_NaL_, I_NaK_, [Ca^2+^]_SR_, [Ca^2+^]_SS_, intracellular sodium concentration [Na^+^]_i_ and action potentials (AP) were compared between the normal condition (pH=7.15) and the acidosis condition (pH=6.7). Compared with the normal condition, prolonged AP, increased [Na^+^]_i_ and elevated [Ca^2+^]_SR_ were observed under the acidosis condition **(Supplementary Figure 1)**.


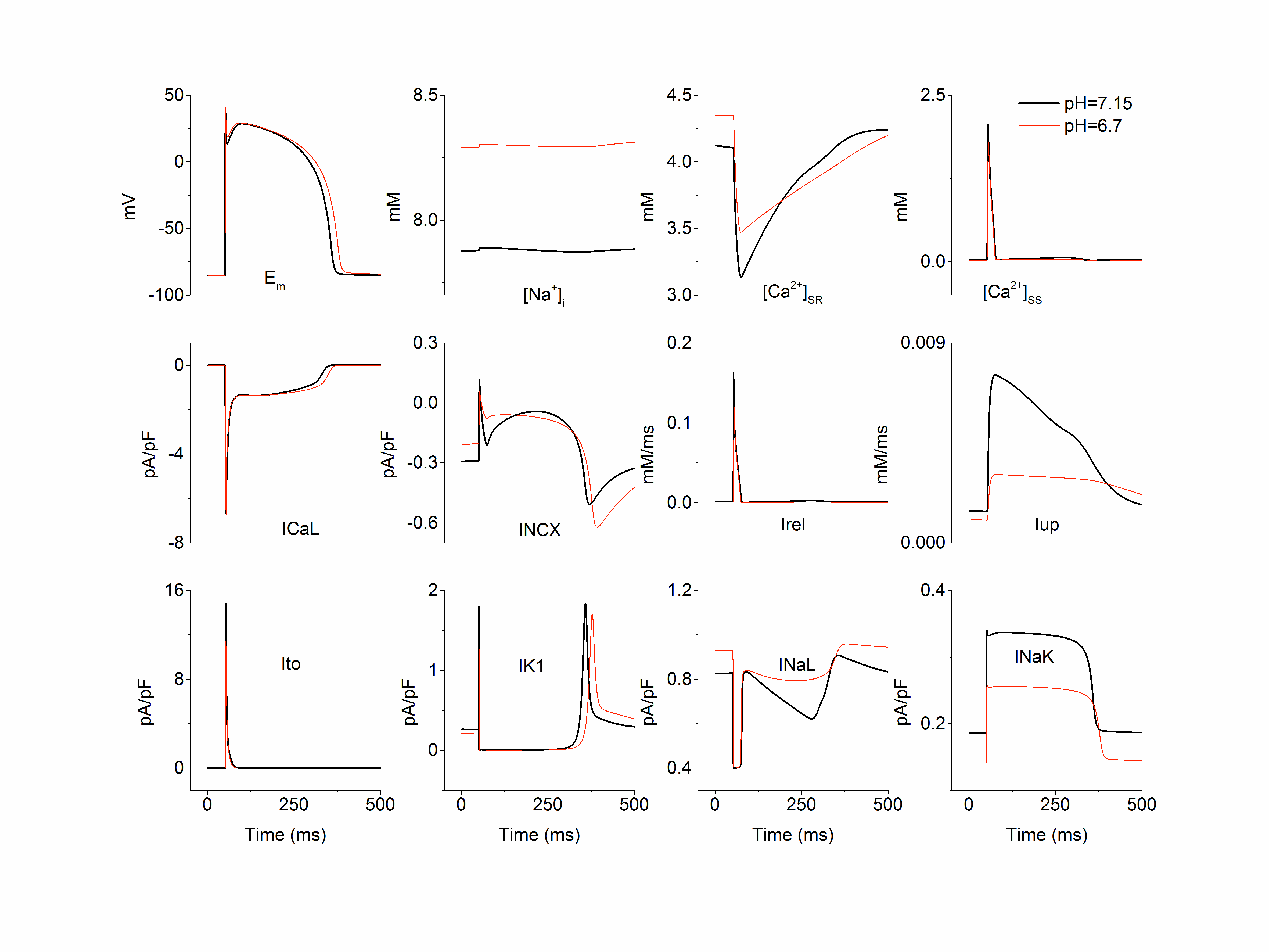


**Supplementary Figure 1.** Action potential (AP), [Na^+^]_i_, [Ca^2+^]_SR_, [Ca^2+^]_SS_, I_CaL_, I_NCX_, I_rel_, I_up_, I_to_, I_K1_, I_NaL_ and I_NaK_ under the normal (pH=7.15) and acidosis (pH=6.7) conditions

# An anisotropic 2D ventricular tissue sheet model

To study the mechanisms by which different pH restoration time periods induce reentrant arrhythmias and to examine the effect of the heterogeneity on the genesis of reentrant arrhythmias, an epicardial 2D tissue of 90 mm ×90 mm, which includes a normal zone (NZ) and a circular central acidotic zone (CZ) of 30 mm in diameter surrounded by a ring-shaped acidotic border zone (BZ) spanning a surface of 45 mm in diameter, was developed (**Supplementary Figure 2B**). The electrophysiological parameters responding to acidosis is indicated in **Supplementary Figure 2A**. As for the CZ, the values of the parameters affected by acidosis were chosen to correspond to their post acidosis values in single cells. In the acidotic BZ, we allowed all parameters to return to their normal values along linear spatial gradients, as previously suggested by Trenor *et al*(Trenor et al., 2005; Trenor et al., 2007; Romero et al., 2009). In the model, different values for longitudinal and transverse resistances were used so as to achieve anisotropy, giving rise to a 4:1 longitudinal-transverse diffusion coefficient (*D*) ratio in the tissue, as previously suggested by Defauw *et al*(Defauw et al., 2014). For the stimulus protocol used in experimental studies(Lascano et al., 2013), after stabilization (the control condition, pH of 7.15 for 1 min), cardiac cells were acidificated (the acidosis condition, pH of 6.7 for 6 min) and then returned to the control condition (the post acidosis condition, pH of 7.15 for 5 min). Single cells in the stimulus protocol were paced with a constant pacing frequency of 70 beats/min. Three conditions was considered in single cell simulations. Suprathreshold DADs was predicted when pH restoration time period is within 0.15 min **(Supplementary Figure 2C**), subthreshold DADs was obtained when pH restoration time period is between 0.15 min and 4 min (**Supplementary Figure 2D**), no ectopic beats was triggered if pH restoration time period is beyond 4 min (**Supplementary Figure 2E**).


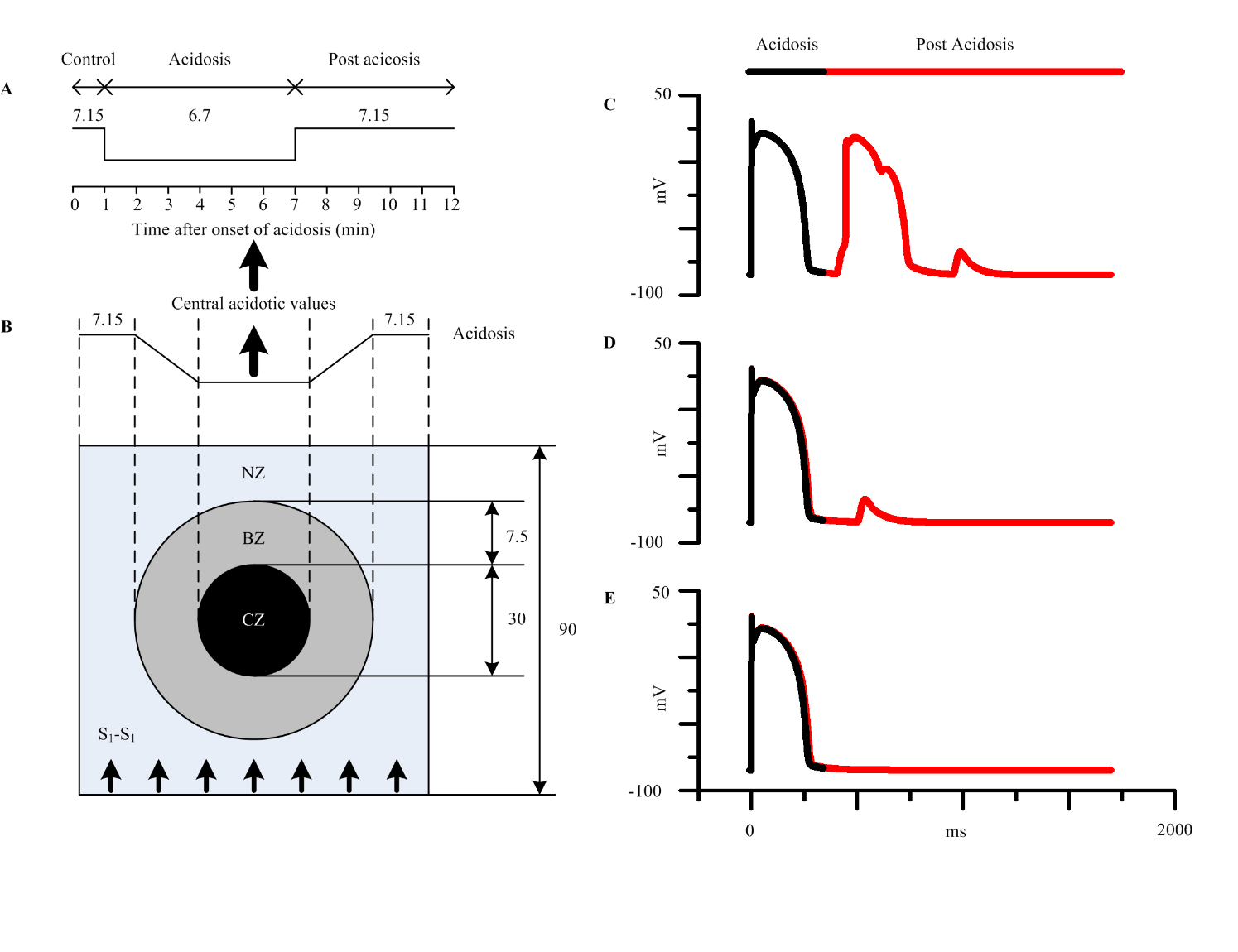


**Supplementary Figure 2.** The anisotropic 2D ventricular tissue sheet model. (**A**) The stimulus protocol used to cells in the acidotic zone (CZ) and the ring-shaped acidotic border zone (BZ) of the 2D tissue. (**B**) The regional acidotic tissue consists of NZ, CZ and BZ. **(C)** Suprathreshold DADs were triggered when the pH restoration time period is within 0.15 min. **(D)** Subthreshold DADs were triggered when the pH restoration time period is between 0.15 min and 4 min. **(E)** No triggered activity occurred when the pH restoration time period is beyond 4 min.





**Supplementary Figure 3.** Snapshots of excitation waves in an anisotropic 2D ventricular tissue sheet. (**A**) The suprathreshold DADs produced a figure-of-eight reentry. No reentry was formed under the subthreshold DADs **(B)** and normal **(C)** conditions.

**
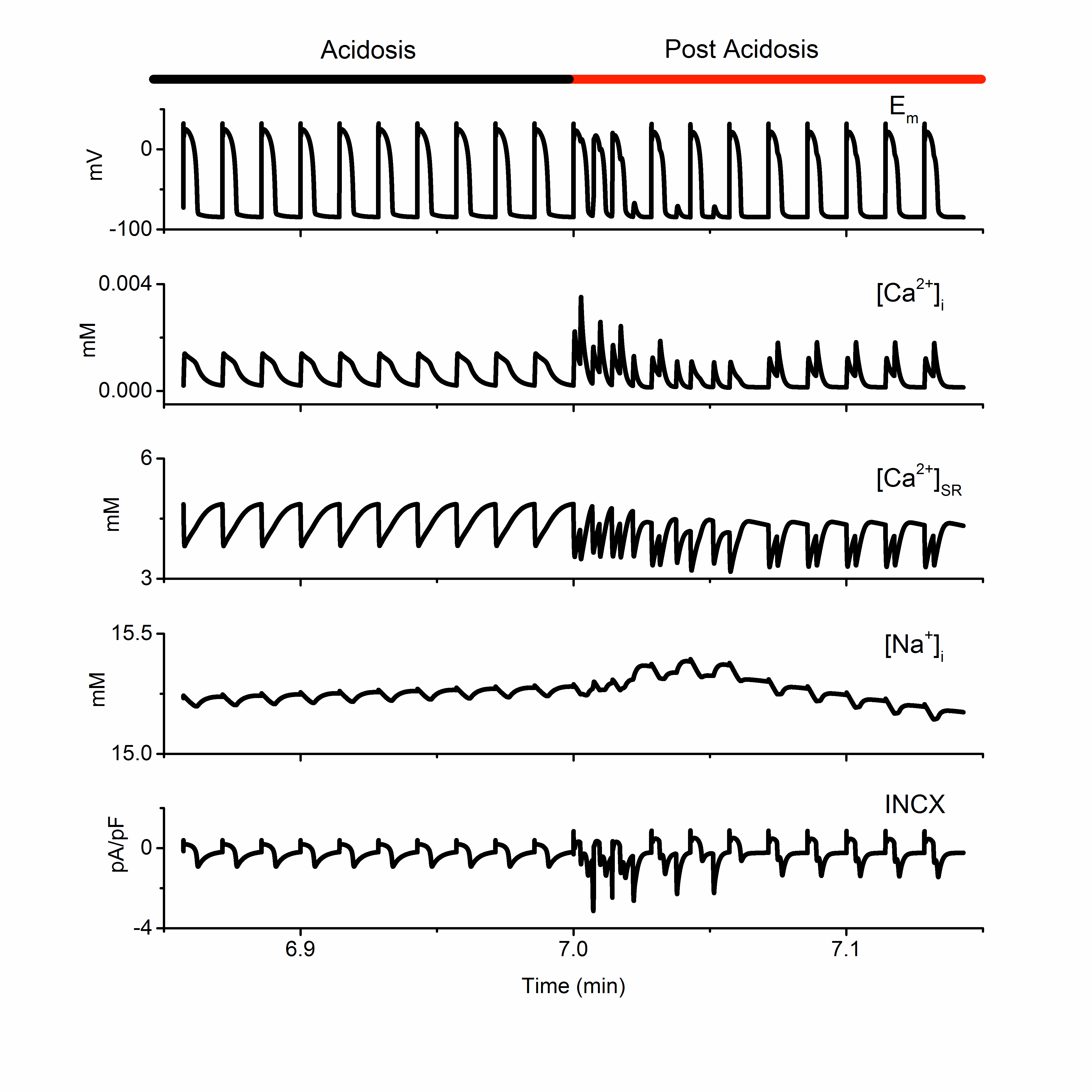
**

**Supplementary Figure 4.** Enlarged records of membrane potentials (E_m_), [Na^+^]_i_, [Ca^2 +^]_i_, [Ca^2 +^]_SR_ and INCX during acidosis and post acidosis. During acidosis, the increase in [Na^+^]_i_ and [Ca^2 +^]_i_ was partly due to acidosis-induced inhibition of NCX. The return to normal pH relieved NCX from acidosis-induced inhibition. The spontaneous SR calcium release resulted from an overloaded SR, through the activation of INCX, which would in turn increase sodium influx, caused the generation of DAD. As pH reached its normal value, [Na^+^]_i_ was still high. This increased [Na^+^]_i_ promoted INCX_,_ enhancing [Ca^2 +^]_i_ and [Ca^2 +^]_SR_ and hence DAD maintenance.

# Supplementary Video Captions

**Supplementary Video 1: Transmural reentry triggered by local sarcoplasmic reticulum (SR) calcium release in post acidosis.** Left: Local SR calcium release failed to induce reentry in normal transmural ventricular wall; Middle: A premature ventricular complex caused by local acidosis stimulation initiated reentry when heterogeneity in the tissue was augmented, but the spiral wave spontaneously terminated; Right: An ectopic activity, originating from regional acidotic epicardium, degenerated into persist reentry when conduction velocity was decreased, resulting in abbreviation of wavelength.

# References

Crampin, E.J., and Smith, N.P. (2006). A dynamic model of excitation-contraction coupling during acidosis in cardiac ventricular myocytes. *Biophysical Journal* 90(9)**,** 3074-3090. doi: 10.1529/biophysj.105.070557.

Decker, K.F., Heijman, J., Silva, J.R., Hund, T.J., and Rudy, Y. (2009). Properties and ionic mechanisms of action potential adaptation, restitution, and accommodation in canine epicardium. *American Journal of Physiology-Heart and Circulatory Physiology* 296(4)**,** H1017-H1026. doi: 10.1152/ajpheart.01216.2008.

Defauw, A., Vandersickel, N., Dawyndt, P., and Panfilov, A.V. (2014). Small size ionic heterogeneities in the human heart can attract rotors. *American Journal of Physiology - Heart and Circulatory Physiology* 307(10)**,** H1456-H1468. doi: 10.1152/ajpheart.00410.2014.

Lascano, E.C., Said, M., Vittone, L., Mattiazzi, A., Mundina-Weilenmann, C., and Negroni, J.A. (2013). Role of CaMKII in post acidosis arrhythmias: A simulation study using a human myocyte model. *Journal of Molecular and Cellular Cardiology* 60**,** 172-183. doi: 10.1016/j.yjmcc.2013.04.018.

O'Hara, T., Virag, L., Varro, A., and Rudy, Y. (2011). Simulation of the undiseased human cardiac ventricular action potential: model formulation and experimental validation. *PLoS Comput Biol* 7(5)**,** e1002061. doi: 10.1371/journal.pcbi.1002061.

Romero, L., Trenor, B., Alonso, J.M., Tobon, C., Saiz, J., and Ferrero, J.M., Jr. (2009). The Relative Role of Refractoriness and Source-Sink Relationship in Reentry Generation during Simulated Acute Ischemia. *Annals of Biomedical Engineering* 37(8)**,** 1560-1571. doi: 10.1007/s10439-009-9721-2.

ten Tusscher, K., and Panfilov, A.V. (2006). Alternans and spiral breakup in a human ventricular tissue model. *American Journal of Physiology-Heart and Circulatory Physiology* 291(3)**,** H1088-H1100. doi: 10.1152/ajpheart.00109.2006.

Trenor, B., Ferrero, J.M., Rodriguez, B., and Montilla, F. (2005). Effects of pinacidil on reentrant arrhythmias generated during acute regional ischemia: A simulation study. *Annals of Biomedical Engineering* 33(7)**,** 897-906. doi: 10.1007/s10439-005-3554-4.

Trenor, B., Romero, L., Ferrero, J.M., Jr., Saiz, J., Molto, G., and Alonso, J.M. (2007). Vulnerability to reentry in a regionally ischemic tissue: A simulation study. *Annals of Biomedical Engineering* 35(10)**,** 1756-1770. doi: 10.1007/s10439-007-9353-3.
